# Balancing feasibility and comprehensiveness: examining medications for reducing emergency hospital admissions

**DOI:** 10.1186/s12916-018-1160-1

**Published:** 2018-10-05

**Authors:** Michael R. Gionfriddo

**Affiliations:** Center for Pharmacy Innovation and Outcomes, Geisinger Precision Health Center, Forty Fort, PA USA

**Keywords:** Emergency admissions, Unplanned admissions, Medications, GRADE, Guidelines, Prioritization, Systematic reviews, Overview of reviews, Umbrella reviews, Search strategy

## Abstract

Emergency hospital admissions are common, with several interventions having been developed to reduce their rates. Bobrovitz et al. summarized the available body of evidence regarding pharmacologic therapies aimed at reducing emergency hospital admissions, and identified 28 medications for which high- or moderate-quality evidence supports their use, 11 of which were identified as being supported by current guideline recommendations. Additionally, the authors identified 28 medications supported by low- or very low-quality evidence, which can serve as targets for future research. The article by Bobrovitz et al. presents a good summary of the evidence, albeit with limitations in the search strategy that cannot guarantee the review as comprehensive. Despite this, the review has important implications for policymakers, guideline panels, researchers, clinicians, and funders since the identified medications can either be targets for quality improvement initiatives or for future research. Bobrovitz et al.’s review highlights the challenge that systematic reviewers face when balancing feasibility and comprehensiveness.

Please see related article: https://bmcmedicine.biomedcentral.com/articles/10.1186/s12916-018-1104-9

## Background

Emergency or unplanned hospital admissions are a common occurrence, with rates varying according to condition (e.g., patients with stroke have a lower rate of emergency admissions than those with heart failure) [1] or medication burden [2], among other factors. Several interventions exist to prevent emergency readmissions, including case management, patient education, home visits, and self-management support [3]. Medications are a core component of these interventions, yet there has been no systematic effort to summarize which medications help to reduce emergency hospital admissions. Bobrovitz et al. [4] aimed to fill this gap by conducting a systematic review of systematic reviews.

Systematic reviews of systematic reviews, also known as overviews of reviews or umbrella reviews, help to summarize the evidence from multiple reviews and are necessary due to the limited scope of existing reviews [5]. For example, a review may be focused on a single medication or condition, but there are many conditions for which a possible outcome could be emergency hospitalization. Thus, to identify interventions likely to reduce the incidence of emergency hospitalizations, Bobrovitz et al. [4] performed an umbrella review searching Medline, PubMed, the Cochrane Database of Systematic Reviews, the Cochrane Database of Abstracts and Reviews of Effects, Google Scholar, and the websites of ten national funding agencies and health charities in the UK for systematic reviews of randomized trials published in English, including reviews that searched two or more databases, assessed study quality, and examined the effect of any medication on emergency hospitalizations among individuals aged 16 years or older. The authors prioritized the medications based upon the strength of evidence as defined by the Grading of Recommendations Assessment, Development and Evaluation (GRADE) Working Group. In addition, they correlated their findings with treatment recommendations found in clinical guidelines from the National Institute for Clinical Excellence (NICE) and cross-referenced these with select guidelines from Europe and America. The review included guidelines that covered heart failure, coronary artery disease, dyslipidemia, ischemic heart disease, asthma, chronic obstructive pulmonary disease, and schizophrenia.

Bobrovitz et al. [4] found 140 reviews that met their criteria, which included 1986 trials; of these, 690 trials and 577,604 patients contributed to the analysis on hospital admission outcomes. The most common populations covered in the reviews were patients with heart failure, asthma, or chronic obstructive pulmonary disease. From the 100 unique medications studied, 28 in 15 patient populations were identified as having high- or moderate-quality evidence supporting their use in reducing emergency hospitalizations as defined by GRADE guidance, whereas 11 were supported by clinical guidelines. These medications included, for example, angiotensin converting enzyme inhibitors for heart failure and statins for stable coronary artery disease.

## A comprehensive search strategy?

While the review by Bobrovitz et al. [4] was generally well conducted, there are several limitations worth mentioning. A systematic review is shaped by its inclusion criteria. By design, the authors may have excluded several studies that may have contained information relevant to the prevention of emergency hospitalizations using medication. While they searched several databases, including some that cover parts of the grey literature, there are several other databases that could have been searched which may have provided useful information, for example, EMBASE, CINAHL, and International Pharmaceutical Abstracts. Although these databases often overlap, several studies have shown that the overlap is far from complete [6, 7]. Similarly, the review only included studies in English and, while there is conflicting evidence regarding language bias [8–10], it remains possible that it missed certain medication interventions which affect emergency hospital admissions. For example, studies related to traditional Chinese medicine are more likely to be indexed in Chinese language databases [10]. Searching the grey literature is an important component of comprehensive systematic reviews [11]; however, Bobrovitz et al. [4] examined sources that were limited to the UK. Expanding the search to international organizations or prominent organizations in other countries may have captured additional studies. Similarly, while the authors sought the input of experts, these were only approached at three conferences in the UK, limiting their reach. Further, for inclusion, studies needed to have searched more than one database and to have assessed and reported on the quality of the included studies. While these are criteria of comprehensive reviews, by excluding studies that failed to assess and report on quality or only searched a single database, some potential interventions may have been excluded. Finally, in addition to the limitations noted above, the authors note in their discussion that many medications which impact hospital admissions were excluded because the results were reported as a composite outcome.

For the prioritization of medications, Bobrovitz et al. [4] first examined NICE guidelines and then expanded to relevant guidelines from Europe and America. To be considered ‘guideline based’, the medication had to be recommended by NICE and at least one other guideline. However, the list of guidelines they identified was not comprehensive. For example, infliximab was identified as having moderate evidence for the treatment of Crohn’s or ulcerative colitis, but was not flagged as guideline based in this article despite recommendations for use from NICE [12] and the American Gastroenterological Association [13].

## Implications

One of the principles of evidence-based medicine is that decisions should be based on systematic summaries of the body of evidence [14]. This emphasis on systematic reviews, and their placement atop the hierarchy of evidence, reflects the strengths inherent in the design. Systematic reviews help identify the extent of heterogeneity present in a body of evidence (i.e., do all studies show similar estimates of effect?) and, when pooled statistically, can help reduce imprecision in estimates of effect [15]. By conducting a review of reviews, Bobrovitz et al. [4] combined several individual reviews together to answer an important clinical question – which medications can impact emergency hospital admissions? Despite the potential limitations in their search strategy, this was relatively unique, as it was not limited by condition or medication class, and instead serves as a broad overview of pharmacologic interventions. By highlighting those medications that are supported by moderate- and high-quality evidence, the review can serve as a guide for policymakers and other stakeholders in the arena of quality measures. As the authors noted, while 11 of these medications are recognized as important by guideline panels, under-prescribing remains an issue. Guideline panels should review the list of 28 medications that were supported by low- or very low-quality evidence and consider calling for additional research or making conditional recommendations, if this is not already done. Emergency hospital admissions are one of many outcomes from which a recommendation could be crafted. If emergency hospital admissions were rated as a critical outcome for a guideline panel, then most situations would call for a conditional recommendation to use (or not) a given medication [16]. Therefore, a conditional recommendation uses language such as ‘might’ rather than ‘should’ [17], thus reflecting limitations in the evidence but also recognizing the potential of the intervention. While additional evidence may not change the confidence in the evidence nor the recommendation from guideline panels, funders could use the list of medications supported by low- or very low-quality evidence to craft specific calls for research for which trials could be specifically developed to contribute to this evidence base.

## Conclusion

Systematic reviews are systematic summaries of a body of evidence that can help a variety of stakeholders make informed decisions, including helping policymakers to craft quality metrics, aiding researchers and funders to decide which research to pursue, or assisting clinicians to decide which therapy to administer. However, systematic reviews are a product of the methods used to create them. When limitations are present in the search strategy, the result could be a systematic summary of the body of evidence that may not be comprehensive. Bobrovitz et al.’s [4] review highlights the challenge that systematic reviewers face when balancing feasibility and comprehensiveness.
